# Short-term functional outcome after fast-track primary total knee arthroplasty: analysis of 623 patients

**DOI:** 10.1080/17453674.2021.1925412

**Published:** 2021-05-12

**Authors:** Jeroen C Van Egmond, Brechtje Hesseling, Hennie Verburg, Nina M C Mathijssen

**Affiliations:** Department of Orthopaedics, Reinier Haga Orthopedisch Centrum, Zoetermeer, the Netherlands

## Abstract

Background and purpose — Early functional outcome after total knee arthroplasty (TKA) has been described before, but without focus on the presence of certain functional recovery patterns. We investigated patterns of functional recovery during the first 3 months after TKA and determined characteristics for non-responders in functional outcome.

Patients and methods — All primary TKA in a fast-track setting with complete patient-reported outcome measures (PROMs) preoperatively, at 6 weeks, and 3 months postoperatively were included. Included PROMs were Oxford Knee Score (OKS), Knee disability and Osteoarthritis Outcome Score Physical Function Short-Form (KOOS-PS), and EuroQol 5 dimensions (EQ-5D) including the self-rated health Visual Analogue Scale (VAS). Patients with improvement on OKS less than the minimal clinically important difference (MCID) were determined as non-responders at that time point. Characteristics between groups of responders and non-responders in functional recovery were tested for differences: we defined 4 groups a priori, based on the responder status at each time point.

Results — 623 patients were included. At 6 weeks OKS, KOOS-PS, and EQ-5D self-rated health VAS were statistically significant improved compared with preoperative scores. The mean improvement was clinically relevant at 6 weeks for KOOS-PS and at 3 months for OKS. Patient characteristics in non-responders were higher BMI and worse scores on EQ-5D items: mobility, self-care, usual activities, and anxiety/depression.

Interpretation — Both statistically significant and clinically relevant functional improvement were found in most patients during the first 3 months after primary TKA. Presumed modifiable patient characteristics in non-responders on early functional outcome were BMI and anxiety/depression.

Most arthroplasty research has focused on long-term functional outcomes and survival of the prosthesis. These outcomes have frequently been used for quality assessments and performance outcomes of the prosthesis itself.

Because around 20% of patients remain unsatisfied after total knee arthroplasty (TKA) (Baker et al. 2007, Bourne et al. 2010), studying early functional outcome patterns more closely might provide important information to further optimize rehabilitation and patient satisfaction.

In a recent article by van Egmond et al. (2021) 3 distinct recovery trajectories were found after TKA, using preoperative, 6 months, and 12 months postoperative Oxford Knee Scores (OKS), of which 2 trajectories at 6 months had approximately the same trajectory and subsequently diverged. Relatively similar patterns have seen in total hip arthroplasty (THA) (Hesseling et al. 2019).

Several studies on early function, pain, and quality of life outcomes after TKA have been published (Andersen et al. 2009, Larsen et al. 2012, Jakobsen et al. 2014, Castorina et al. 2017, Schotanus et al. 2017, Husted et al. 2021). Moreover, Canfield et al. (2020) concluded that most improvement in function and pain is gained during the first 6 months postoperatively.

Although functional rehabilitation in TKA and THA patients before 6 months has been studied (Van Egmond et al. 2015, Klapwijk et al. 2017), the question remains whether differences in functional recovery patterns exist before the 6-month mark in TKA patients.

We expect that rehabilitation might be further optimized with knowledge of early functional rehabilitation patterns. Therefore, the primary objective of this study was to determine patterns in functional outcome at 6 weeks and 3 months after primary TKA. Secondary objectives were a non-responder analysis and to determine characteristics for non-responders in early functional recovery.

## Patients and methods

This is a retrospective exploratory cohort study. Data, all prospectively collected, was gathered from the digital PROMs database of our institution.

### Patients

As standard procedure in our institution, during the study period from January 2015 to August 2017, all patients with primary TKA were asked to complete PROMs preoperatively, and received digital PROMs questionnaires at 6 weeks and 3 months postoperatively (OnlinePROMs, Amsterdam, the Netherlands).

All patients who underwent primary TKA with fast-track recovery at our institution during the study period were eligible for inclusion. Patients with completed PROMs at all 3 time points were included for analysis. In patients with bilateral TKA during the inclusion period only the results of the first TKA were analyzed.

### Measurements

Included PROMs were OKS, Knee disability and Osteoarthritis Outcome Score Physical Function Short-Form (KOOS-PS), and EuroQol 5 dimensions (EQ-5D-3L).

The EQ-5D-3L questionnaire comprises 5 questions on the dimensions of health, including mobility, self-care, usual activities, pain/discomfort, and anxiety/depression. The second part of the EQ-5D contains a self-rated health score on a visual analogue scale (VAS) from 0 to 100, where 0 represents the worst imaginable health and 100 the best imaginable health. The EQ-5D self-rated health VAS was used from every administration to determine general health improvement (Devlin et al. 2010).

The KOOS-PS score ranges from 0 to 100%, where 0% represents no difficulty in physical functioning (Perruccio et al. 2008). The minimal clinically important difference (MCID) is 4% for KOOS-PS, while a moderate improvement is stated at 32% (Singh et al. 2014).

The OKS is based on 12 questions regarding pain and function of the knee. Total score ranges from 0 to 48 with higher scores indicating better function and less pain (Dawson et al. 1998). Anchor-based methods showed that a change in score of approximately 9 points on the OKS indicates a meaningful improvement at the group level (Beard et al. 2015). Missing data was handled according to the specific questionnaire rules (Murray et al. 2007).

For non-responder analysis we used the OKS, mainly to ensure our results could be compared with our previous study. Moreover, we find the OKS to cover a broader range of functional outcome than the KOOS-PS. Patients were rated as responders based on MCID of the OKS; an improvement on OKS above the MCID of 9 points labelled patients as responders. Both at 6 weeks and 3 months improvement was determined. Consequently 4 groups were formed including: (1) responder at 6 weeks, responder at 3 months; (2) non-responder at 6 weeks, responder at 3 months; (3) non-responder at 6 weeks, non-responder at 3 months; and (4) responder at 6 weeks, non-responder at 3 months.

### Statistics

Normally distributed outcomes were presented as mean and 95% confidence interval (CI). Not normally distributed outcomes were presented as median, total, and interquartile range (IQR).

Repeated-measures ANOVA was used to determine changes in outcome over time for OKS, KOOS-PS, and EQ-5D self-rated health VAS separately, using all 3 time points. If there was a statistically significant change over time, a priori planned post-hoc ANOVA analysis was performed to compare preoperative scores with 6 weeks, and scores at 6 weeks with 3 months to determine at which point in time the scores improved (Twisk 2003).

For responder analysis only the OKS was used to determine whether a patient was a responder. Groups of responders and non-responders were compared and tested for differences on their characteristics using chi-square, Kruskal–Wallis, and ANOVA. If there was an overall statistically significant difference between the groups, a priori planned post-hoc Mann–Whitney U analysis with Bonferroni correction was performed to test which groups differed.

Characteristics of interest were dichotomized for analysis; age (≤ 75 vs. > 75), ASA (class I–II vs. III–IV), and EQ-5D scores (no problems vs. moderate-to-severe problems).

For statistical analyses IBM SPSS statistics version 25 (IBM Corp, Armonk, NY, USA) was used. A p-value of 0.05 or lower was considered statistically significant.

### Ethics, funding, and potential conflicts of interest

This study did not fall under the scope of the research with human subjects Act according to the local ethical committee as this study placed no additional burden on the patient. This study was conducted according to the Declaration of Helsinki (version 64, October 2013). No funding was received for this study. The authors have no conflicts of interest to declare.

## Results

623 patients with unilateral primary TKA in a fast-track setting were included (Table 1). Median age was 70 years, and 420 (67%) patients were female. 437 patients (70%) were classified as ASA 2. Median BMI was 29 (IQR 26–36) for the total group.

**Table 1.** Patient demographics (N = 623). Values are count (%) unless otherwise specified

Demographics	623 TKA
Age, median [IQR] (range)	70 [64–77] (32–93)
Female sex	420 (67)
Smoking yes	64 (10)
ASA score	
I	100 (16)
II	437 (70)
III	86 (14)
BMI	
Normal weight (< 25)	100 (16)
Overweight (25–30)	267 (43)
Obesity (> 30)	256 (41)
LOS, median [IQR] (range)	2 [2–3] (0–9)

LOS = length of stay by hospital nights.

### Primary outcome

Both the function scores (OKS and KOOS-PS) and EQ-5D self-rated health VAS improved during the first 3 months as presented in Table 2. Since all scores were not normally distributed the median, total range, and IQR were presented.

**Table 2.** Median function scores at the 3 time points. Values are median [IQR] (range)

Item	Preoperative	6 weeks	3 months
OKS	23 [17–28] (2–45)	30 [25–36] (4–48)^a^	35 [29–41] (9–48)^b^
KOOS-PS	51 [42–62] (15–100)	40 [34–46] (0–100)^a^	35 [28–44] (0–100)^b^
EQ-5D VAS	71 [60–81] (3–100)	75 [60–86] (0–100)^a^	79 [65–88] (6–100)^b^

a Significant improvement between preoperative and 6 weeks, Wilks’ λ < 0.001.

b Significant improvement between 6 weeks and 3 months, Wilks’ λ < 0.001.

Repeated-measures ANOVA of both function scores and EQ-5D self-rated health VAS over the 3-month postoperative period showed statistically significant improvement. For OKS, KOOS-PS, and EQ-5D self-rated health VAS the Wilks’ λ was < 0.001 for the preoperative to 6 weeks period and from 6 weeks to 3 months as well.

**Figure 1.**
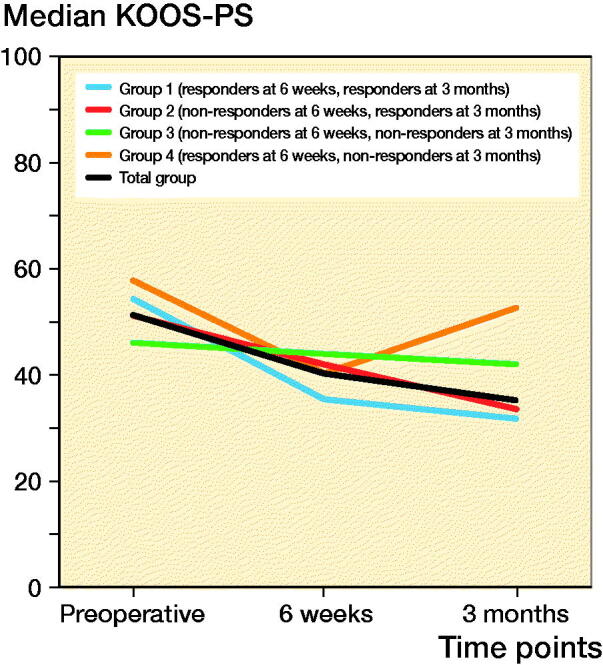
Median KOOS-PS course in 3 months.

The improvement on KOOS-PS at 6 weeks postoperatively was 11%, which is clinically relevant. At 3 months, compared with preoperatively, an improvement of 16% was found (Figure 1).

**Figure 2.**
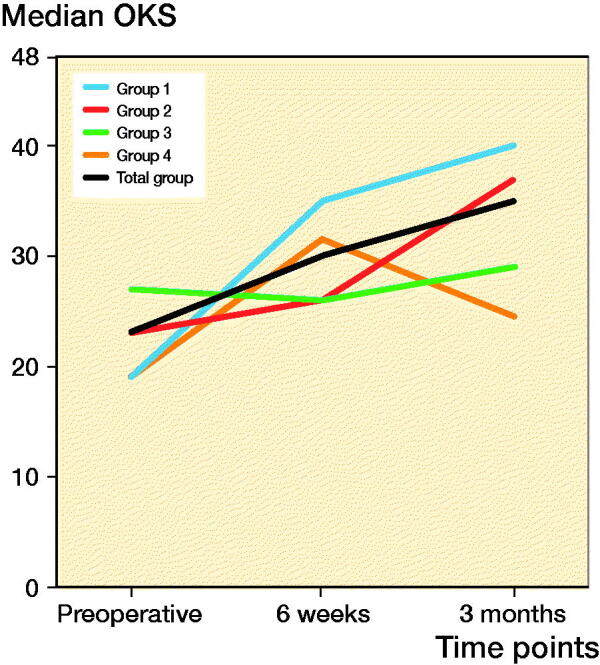
Median OKS course in 3 months.

The OKS improved 7 points during the first 6 weeks, which is statistically significant but not clinically relevant. At 3 months a statistically significant and clinically relevant improvement of 12 points was found (Figure 2).

**Figure 3.**
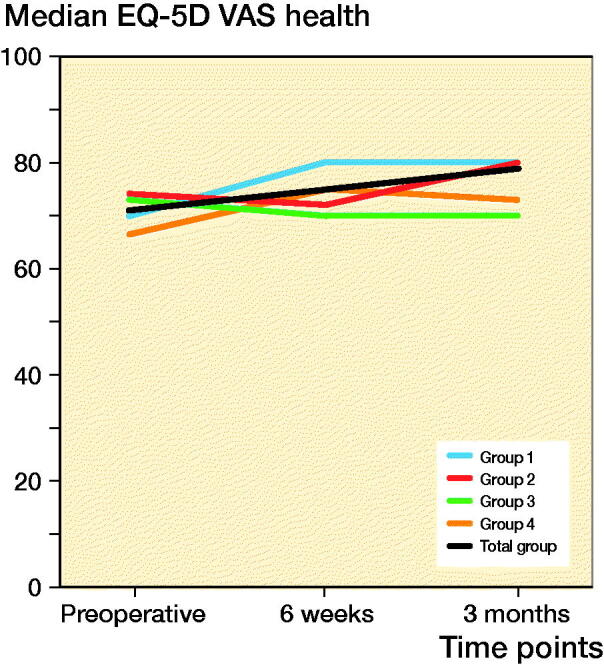
Median EQ-5D VAS health course in 3 months.

The EQ-5D self-rated health VAS showed improvement both at 6 weeks and 3 months postoperatively, of respectively 4 and 8 points compared with preoperative levels (Figure 3).

### Secondary outcome

Responder analysis was performed based on MCID of OKS at 6 weeks and 3 months postoperatively, compared with preoperative scores. The percentage of responders improved from 44% at 6 weeks to 67% at 3 months.

The predefined 4 groups comprised: (1) responder at 6 weeks, responder at 3 months (41%); (2) non-responder at 6 weeks, responder at 3 months (23%); (3) non-responder at 6 weeks, non-responder at 3 months (33%); and (4) responder at 6 weeks, non-responder at 3 months (3%).

Groups 1 and 2 were determined as responders versus groups 3 and 4 as non-responders.

There was a statistically significant difference between the groups regarding BMI and EQ-5D items: mobility, self-care, usual activities, and anxiety/depression (Table 3). In the planned post-hoc analysis groups 1 and 2 were mostly comparative (Table 4). On EQ-5D anxiety/depression, group 4 differed from the other groups (Table 4). Finally, the distribution of normal and high BMI was different between groups 1 and 2 compared with group 4 (Table 3). The post-hoc pairwise analysis presented in Table 4 showed a statistically significant difference between groups 3 and 4 for BMI.

**Table 3.** Responder analysis. Values are count (%) unless otherwise specified

Factor		Group 1	Group 2	Group 3	Group 4	
Total number		218 (41)	119 (23)	177 (33)	14 (3)	
Age	≤ 75 years	142 (65)	90 (76)	121 (68)	10 (71)	0.3^a^
	> 75 years	76 (35)	29 (24)	56 (32)	4 (29)	
BMI	< 25	26 (12)	16 (13)	42 (24)	1 (7)	0.02^b^
	25–30	101 (46)	55 (46)	71 (40)	3 (21)	
	> 30	91 (42)	48 (40)	64 (36)	10 (71)	
Smoking	Yes	21 (10)	11 (9)	19 (11)	1 (7)	1.0^a^
	No	197 (90)	108 (91)	158 (89)	13 (93)	
ASA	I–II	197 (90)	102 (86)	155 (88)	11 (79)	0.4^b^
	III–IV	21 (10)	17 (14)	22 (12)	3 (21)	
Preoperative EQ-5D						
Mobility						
No problems		8 (4)	2 (2)	18 (10)	0 (0)	0.004^b^
Some problems in walking or confined to bed		210 (96)	117 (98)	159 (90)	14 (100)	
Self-care						
No problems		165 (76)	101 (84)	151 (85)	7 (50)	0.001^b^
Some problems or unable to wash or dress		53 (24)	18 (15)	26 (15)	7 (50)	
Usual activities						
No problems		32 (15)	18 (15)	50 (28)	0 (0)	0.001^b^
Some problems or unable to perform usual activities		186 (85)	101 (85)	127 (72)	14 (100)	
Pain/discomfort						
No pain or discomfort		16 (7)	10 (8)	25 (14)	1 (7)	0.1^b^
Moderate or extreme pain or discomfort		202 (93)	109 (92)	152 (86)	13 (93)	
Anxiety/depression						
Not anxious or depressed		173 (79)	91 (76)	137 (77)	4 (29)	< 0.001^b^
Moderate or extremely anxious or depressed		45 (21)	28 (24)	40 (23)	10 (71)	
VAS health, median		70	74	73	67	0.3^c^
IQR		60–81	60–82	60–82	50–79	
range		11–100	25–100	13–100	3–100	

Group 1: responders at 6 weeks, responders at 3 months.

Group 2: non-responders at 6 weeks, responders at 3 months.

Group 3: non-responders at 6 weeks, non-responders at 3 months.

Group 4: responders at 6 weeks, non-responders at 3 months.

a Chi-square

b Kruskall–Wallis

c ANOVA

**Table 4.** Post-hoc pairwise analysis

Item	Group 1 vs. 2	Group 1 vs. 3	Group 1 vs. 4	Group 2 vs. 3	Group 2 vs. 4	Group 3 vs. 4
BMI	1.0	0.2	0.4	0.8	0.3	0.05
EQ-5D						
Mobility	1.0	0.03	1.0	0.008	1.0	0.6
Self-care	0.3	0.1	0.1	1.0	0.01	0.008
Usual activities	1.0	0.004	1.0	0.03	1.0	0.06
Anxiety/depression1.0	1.0	< 0.001	1.0	< 0.001	< 0.001	

Group 1–4: See Table 3.

The median OKS in group 1 improved from 19 preoperatively to 40 at 3 months and group 2 improved from 23 to 37 (Table 5). This is in contrast to groups 3 and 4 where median OKS at 3 months showed only minimal improvement from 27 preoperatively to 29 at 3 months for group 3, and 19 to 25 for group 4 (Table 5).

**Table 5.** OKS per group for each time-point. Values are median [IQR] (range)

Group	Preoperative	6 weeks	3 months
1	19 [14–24] (3–36)	35 [30–40] (12–48)	40 [34–44] (21–48)
2	23 [19–27] (3–37)	26 [22–31] (11–42)	37 [34–42] (16–48)
3	27 [22–31] (5–45)	26 [21–31] (5–46)	29 [24–34] (9–47)
4	19 [16–25] (9–28)	32 [27–36] 19–39)	25 [20–28] (15–33)

Group 1–4: See Table 3.

## Discussion

The primary goal of this study was to determine patterns in early functional outcome after primary TKA. The most important finding was the statistically significant and clinically relevant early improvement of both function scores at 6 weeks and 3 months postoperatively for the sample as a whole. Moreover, we examined 4 a priori defined subgroups. Patient characteristics for non-responders were higher BMI and worse scores on EQ-5D items: mobility, self-care, usual activities, and anxiety/depression

With the knowledge that subgroups in TKA recovery exist, based on this study and previous studies, we have to use this knowledge to further improve rehabilitation and outcomes. For example, expectation management can be used in patients at risk of non-responding. Recently, preoperative education and expectation modification was found to increase fulfillment of expectations and concomitant higher satisfaction (Tolk et al. 2021). Therefore more individual rehabilitation might be needed instead of the usual generic type. Preoperative education and the outpatient physical therapist might play a major role in this, as patients are admitted to the hospital relatively soon after this.

In addition, further studies are needed on how to identify non-responding patients preoperatively and provide better selection criteria. Further research is also needed to find what will help non-responding patients preoperatively and during the postoperative rehabilitation. There might, for example, be a need for more support or guidance in the rehabilitation by a physical therapist.

Besides the improvement on both function scores, there was also a statistically significant improvement in EQ-5D self-rated health VAS. This is in line with the findings of Larsen et al. (2012), who found improved health-related quality of life scores in knee arthroplasty patients with no or mild pain and good function. To the best of our knowledge, no MCID has been determined for EQ-5D self-rated health VAS, therefore it is unknown whether the improvement was clinically relevant as well. In this study the EQ-5D self-rated health VAS was used instead of the index score of the EQ-5D, because we were not interested in estimating quality-adjusted life years (QALYs). Moreover, index scores are not comparable internationally, as converting EQ-5D to an index score is referenced nationally.

Our findings were also in accordance with Husted et al. (2021), who found a median OKS 3 months postoperatively of 32 and 31 in the group discharged on day of surgery and not discharged on day of surgery, respectively. We found in our analysis of fast-track TKA patients a median OKS at 3 months of 35 (IQR 29–41).

We used the OKS for non-responder analysis, as this validated score was previously used in the study by van Egmond et al. (2021). Therefore our results would be more easily compared with the results from that study. Moreover, we find the OKS covers a broader range of functional outcome than the KOOS-PS. Characteristics of interests were dichotomized including age (≤ 75 vs. >75), ASA (1–2 vs. 3–4), and EQ-5D (no problems vs. moderate-to-severe problems), and BMI was divided into 3 groups, to prevent small group sizes in analysis.

The post hoc pairwise analysis in Table 4 shows a statistically significant BMI between groups 3 and 4. However, Table 3 presents an obvious difference in percentages of high and normal BMI between groups 1 and 2 compared with group 4. Even though these differences did not reach statistical significance, we find these differences large enough to be of clinical relevance.

In our non-responder analysis, non-responders differed on the EQ-5D items mobility, self-care, usual activities, and anxiety/depression, compared with the other groups. In several studies, poor mental health is related to poor functional outcome (Sorel et al. 2019, Melnic et al. 2021, Hafkamp et al. 2021). Other studies are less distinct and did not find a relationship between anxiety and suboptimal outcomes (Wood et al. 2021). Nevertheless, previous studies showed that psychological support might lead to lower incidence of pain, anxiety/depression, and improve faster recovery (Tristaino et al. 2016, Sorel et al. 2020). Preoperative analysis of the presence of these factors and concomitant treatment might be an effective way to improve the satisfaction rate of TKA. Currently we perform no preoperative screening for psychological status in our institution. This might be feasible with the Pain Catastrophizing Scale (PCS) or Hospital Anxiety and Depression Scale (HADS) (Mercurio et al. 2020). The recently published systematic review by Sorel et al. is promising and described various interventions with good effect on postoperative pain, quality of life, and function for psychological distress in TKA patients (Sorel et al. 2020). Therefore further studies are needed to identify these patients preoperatively and to examine in which way adequate therapy can be provided in this setting.

A major strength of this study is the relatively large number of included patients. However, there are some limitations of this study.

The most important is the retrospective design with all its known forms of bias. However, all data was collected prospectively and validated questionnaires have been used.

Furthermore, the results were based on single institutional data, which might make the results less generalizable. Given that our results were comparable to previously published studies from other countries, we feel this is a minor limitation.

First multinomial logistic regression analysis was performed to test for patient characteristics in the 4 determined groups. Because errors occurred due to small group sizes, these analyses were not valid. Therefore, descriptive statistics were performed resulting in a more exploratory study. No causal relations can be drawn from our non-responder analysis. However, this is the first study that presents patterns in early functional outcome after TKA. New studies are needed to confirm and further define our findings.

We used PROMs to determine early functional recovery after TKA. Previous studies concluded that improvement in PROMs does not correlate with objectively assessed function (Luna et al. 2017, Fransen et al. 2019). We are aware that our findings based on PROMs might not fully represent objective function. However, as the subjective PROMs relate to how patients themselves experience their function, we regard this as a highly valuable outcome.

Finally, no PROMs data was available at further time points up to 1 year to present a detailed course of functional outcomes during the first postoperative year.

In conclusion, orthopedic surgeons and patients can expect improved functional outcomes early after TKA surgery at 6 weeks postoperatively and substantial improvement at 3 months. Concomitant health status improvement was detected as well in this early postoperative phase. Modifiable patient characteristics for non-responders on early functional outcome were BMI and anxiety/depression. Preoperative treatment of these factors might improve postoperative outcomes.
